# Biomaterials‐Involved Construction of Extracellular Matrices for Tumor Blockade Therapy

**DOI:** 10.1002/EXP.20240229

**Published:** 2025-03-16

**Authors:** Jinfeng Sun, Yang Liu, Jingshan Sun, Jianxun Ding, Xuesi Chen

**Affiliations:** ^1^ Key Laboratory of Polymer Ecomaterials Changchun Institute of Applied Chemistry, Chinese Academy of Sciences Changchun P. R. China; ^2^ College of Chemistry Jilin University Changchun P. R. China; ^3^ School of Applied Chemistry and Engineering University of Science and Technology of China Hefei P. R. China; ^4^ Institute of Bioengineering, École Polytechnique Fédérale de Lausanne (EPFL) Lausanne Switzerland

**Keywords:** artificial extracellular matrix, biomineralization, fibrogenesis, gelation, tumor blockade therapy

## Abstract

Extracellular matrices (ECMs) play a crucial role in the onset and progression of tumors by providing structural support and promoting the proliferation and metastases of tumor cells. Current therapeutic approaches targeting tumor ECMs focus on two main strategies: Inhibiting matrix degradation to prevent metastases and facilitating matrix degradation to enhance the penetration of drugs and immune cells. However, these strategies may lead to unintended consequences, such as tumor growth promotion, drug resistance, and side effects like fibrotic changes in healthy tissues. Biomaterials have made significant progress in fabricating artificial ECMs for tumor therapy by inducing biomineralization, fibrogenesis, or gelation. This perspective explores the fundamental concepts, benefits, and challenges of each technique. Additionally, future improvements and research directions in artificial ECMs are discussed, highlighting their potential to advance tumor therapy.

## Graphical abstract

Extracellular matrices (ECMs) play a crucial role in tumor growth and metastases by providing structural support and facilitating tumor cell proliferation. In this review, we explore the development of artificial ECMs engineered through biomineralization, fibrogenesis, and gelation. These bioinspired ECMs create a protective barrier around tumor cells, restricting substance exchange, inhibiting metastases, and potentially inducing tumor cell death, which offers advanced strategies for tumor therapy.

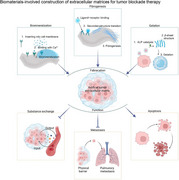

## 1 Introduction

The extracellular matrices (ECMs) are intricate networks composed of structural proteins, such as collagen and elastin, along with glycosaminoglycans, proteoglycans, and cell adhesion molecules like fibronectin and laminin [1]. These structures provide essential mechanical and biochemical support to adjacent cells, promoting cell proliferation and differentiation and maintaining tissue integrity [1, 2]. Within the tumor microenvironments (TMEs) [3], changes in protein composition and increased molecular cross‐linking result in higher matrix density in tumors [4]. These transformations modify the biochemical, biomechanical, and structural characteristics of ECMs, reshaping the TMEs to support tumor proliferation [4, 5, 6].

ECMs contribute to tumor metastases by storing and releasing growth factors, such as vascular endothelial growth factor (VEGF), transforming growth factor‐*β* (TGF‐*β*), and fibroblast growth factor (FGF). These factors influence signaling pathways through interactions between integrins and ECM proteins, transmit mechanical signals, including tissue stiffness, and create an immunosuppressive microenvironment [7]. Moreover, matrix metalloproteinases (MMPs) facilitate tumor metastases by degrading the components of ECMs [8]. Given the role of changes in tumor ECMs in advancing tumor processes, targeting ECMs has become a pivotal direction in contemporary tumor treatment research [7].

Currently, strategies to regulate tumor ECMs aim to either inhibit degradation to prevent metastases or enhance degradation to improve the penetration of drugs and immune cells. Regarding the suppression of degradation, MMP inhibitors block ECM breakdown. However, MMPs are also essential in normal tissues, and their inhibition may lead to adverse effects, such as joint pain, fibrosis, and further tissue damage [9, 10]. Additionally, tumor cells circumvent MMP inhibition by activating alternative pathways or upregulating other proteolytic enzymes [11]. Conversely, ECM degradation may be enhanced using enzymes, such as collagenase [12] and hyaluronidase [13], or by inhibiting the synthesis and maturation processes of ECMs‐essential proteins. For instance, lysyl oxidase inhibitors prevent the cross‐linking of collagen and elastin fibers [14, 15, 16], but excessive ECM degradation poses risks, which release growth factors and cytokines anchored in the ECMs, triggering inflammatory signals and potentially promoting tumor progression [17]. Further, weakening tumor ECMs potentially enhances tumor migration and invasion [18].

**SCHEME 1:**
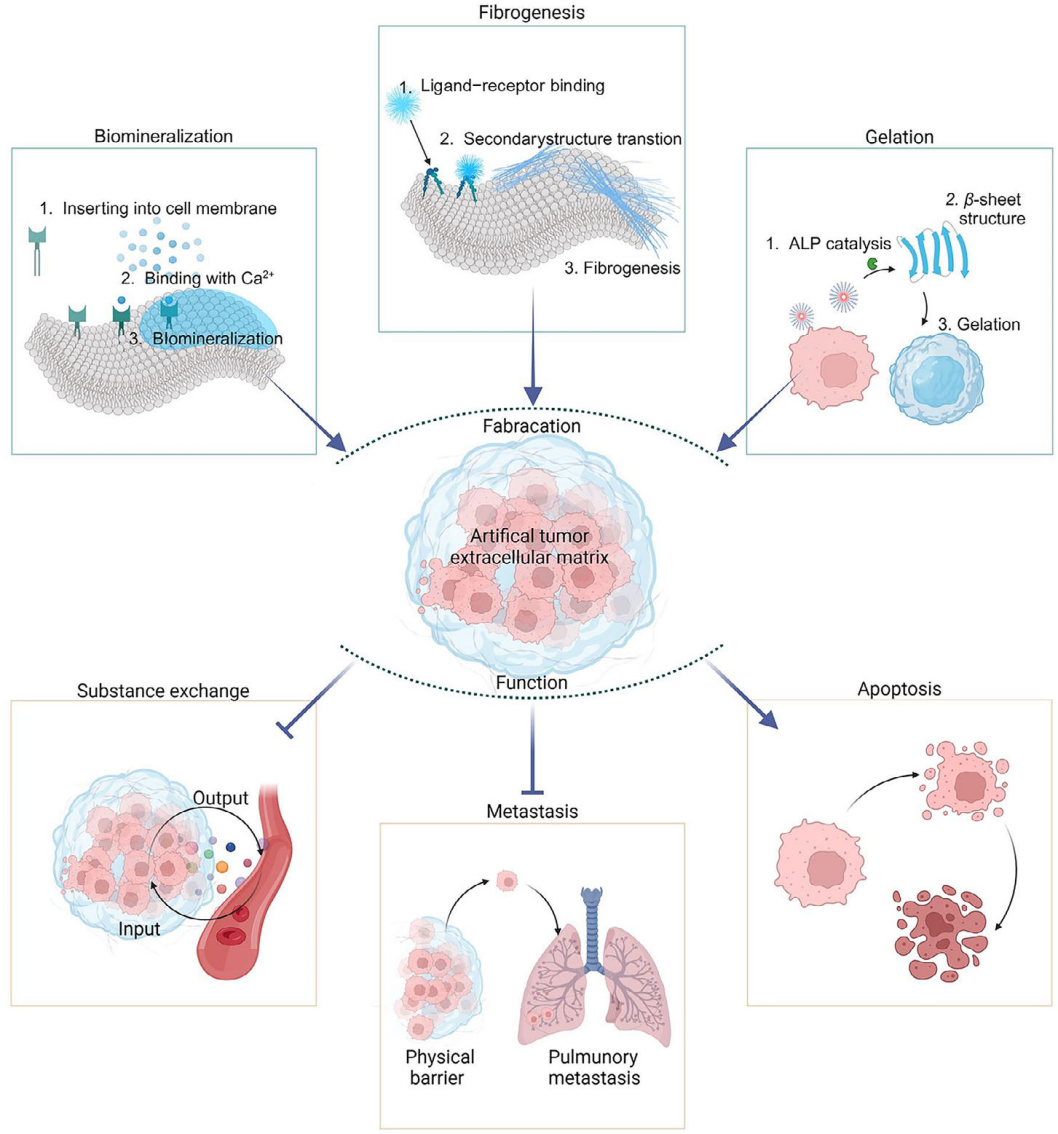
Construction of artificial tumor ECMs utilizing biomineralization, fibrogenesis, and gelation strategies. These ECMs form a barrier around tumor cells, restricting substance exchange, impeding metastases, and potentially inducing apoptosis to inhibit tumor progression.

To overcome these limitations, biomaterials with excellent biocompatibility, tunable structural morphology, and responsiveness to the TMEs provide a versatile platform to control the physical and biochemical properties of ECMs in tumor therapy [19]. Biomaterials play a crucial role in tumor blockade therapy by constructing artificial tumor ECMs that serve as exogenous barriers around tumor cells, restricting substance exchange, impeding metastases, and potentially inducing apoptosis. By serving as both physical and biochemical barriers, biomaterials inhibit the migration of tumor cells and prevent them from invading surrounding tissues, thus reducing the risk of metastases. This review categorizes artificial tumor ECMs based on their distinct formation mechanisms, including biomineralization, fibrogenesis, and gelation (Scheme 1). The fundamental concepts, construction methods, advantages, and challenges of each mechanism are discussed.

## 2 Biomineralization

Biomineralization is a biological process in which cells produce and deposit inorganic minerals, such as calcium (Ca), silicon (Si), iron (Fe), and phosphate (P) [20]. This process typically involves nucleation on organic matrices, such as proteins and polysaccharides, resulting in diverse structures, including bones, teeth, and seashells [21]. The specific morphologies of these mineralized structures, such as their sizes, shapes, and crystallinity, are heavily influenced by the properties of organic matrices and the controlled intracellular microenvironments in which mineral nucleation and subsequent growth occur. The fundamental biomineralization process involves nucleation on organic matrices, such as proteins and polysaccharides, which localize mineral‐related ions at specific binding sites [20, 22–24]. Once nucleation begins, minerals, such as Ca and P, gradually accumulate, forming crystals that grow over time [23]. These structures can be remodeled as needed depending on the biological requirements of the organism [25]. These mineralized biomaterials exhibit many morphologies, from simple prismatic crystals to complex hierarchical architectures, which provide remarkable mechanical properties, such as strength and toughness. Biomineralization occurs through highly controlled cell activities that regulate the precise localization and deposition of minerals, generating biomaterials with remarkable structural properties [26]. Understanding the mechanisms of biomineralization holds great potential for the development of biomaterial science, particularly for developing biomaterials that mimic or harness these natural processes for medical applications [27, 28, 29].

**FIGURE 1:**
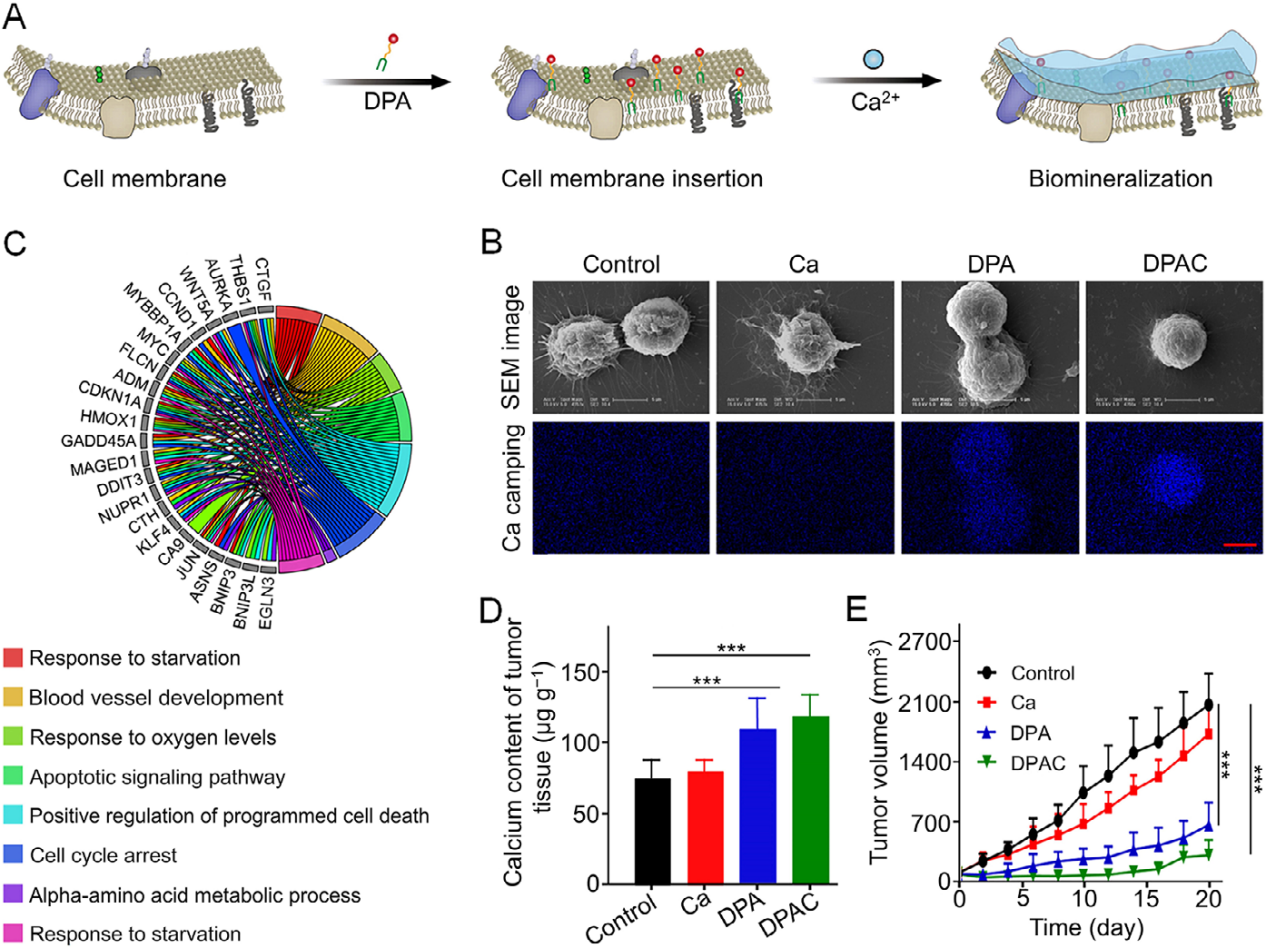
Constructing artificial tumor ECMs using a biomineralization strategy. (A) Biomineralization. (B) SEM images and Ca mapping analysis of 143B cells. Scale bar = 5 µm. (C) Circos plot depicted the subordination between the 23 representative DEGs and their enriched pathways. (D) Ca contents of tumor tissues. (E) Growth curves of tumors after treatment with normal saline as Control, Ca, DPA, or DPAC. All statistical data are represented as mean ± standard deviation (SD; *n* = 5; *P < 0.05, **P < 0.01, ***P < 0.001). Reproduced with permission [30]. Copyright 2022, John Wiley & Sons.

Drawing inspiration from natural biomineralization, Ding et al. developed a controllably synthesized polymer, 1,2‐distearoyl‐*sn*‐glycero‐3‐phosphoethanolamine‐*N*‐poly(ethylene glycol)‐alendronate (DSPE‐PEG‐ALN, DPA) [30]. This polymer was engineered to induce the formation of a mineralized shell around osteosarcoma cells. The DSPE component, with its structural similarity to cell membrane, facilitated its integration into tumor cell membrane. Moreover, the ALN segment, containing bisphosphonate group, had a high affinity for calcium ion (Ca^2+^), promoting biomineralization around tumor cells. DPA effectively targeted and encapsulated osteosarcoma cells, forming a mineral layer (Figure 1A,B). The stiffness of the cells, measured by Young's modulus, increased by 1.40 and 2.41 times in the DPA and DPAC (DPA+Ca) groups, respectively, compared to that of the Control group, demonstrating that the mineral shell enhanced cell rigidity. Upon administration, DPA integrated into cell membrane and initiated continuous mineral deposition, creating a robust physical barrier around the tumor. The RNA sequencing‐based differentially expressed genes (DEG) analysis results revealed that this mineralized barrier significantly reduced the exchange of critical nutrients and biochemical signals, which strongly inhibited the growth of primary osteosarcoma and further tumor progression (Figure 1C). Notably, this approach resulted in a tumor suppression rate of 73.2%, indicating its potential effectiveness in clinical applications for osteosarcoma (Figure 1D,E).

Despite these promising results, the non‐selective characteristic of DSPE integration into cell membrane raised concerns regarding the unintended mineralization of healthy cells. To improve targeting specificity, Ding et al. developed biomineralization‐inducing nanoparticle (BINP) composed of dodecylamine‐poly((*γ*‐dodecyl‐l‐glutamate)‐*co*‐(L‐histidine))‐*block*‐poly(L‐glutamate‐*graft*‐alendronate), which was particularly reactive to the typical acidic microenvironments of tumor site [31]. This property enabled BINP to expand and expose its long aliphatic chain, increasing the probability of inserting into tumor cell membrane. Once integrated, the bisphosphonate groups on the cell surface facilitated CaP deposition around tumor cells, effectively isolating them. The atomic force microscope (AFM) results showed a significant increase in Young's modulus of osteosarcoma cells treated with BINP, with stiffness increasing by 1.55‐fold at pH 7.4 and 3.99‐fold at pH 6.5, indicating a stiffening of the cell membrane due to mineralization. The half‐maximal inhibitory concentration (IC_50_) of BINP on osteosarcoma cells was 0.75 mg mL^−1^ at pH 7.4 and 0.49 mg mL^−1^ at pH 6.5 after 24 h. In animal models, intravenous administration of BINP to mice with osteosarcomas resulted in tumor inhibition rates of 59.3% in subcutaneous models and 52.1% in orthotopic models. These findings demonstrate that BINP selectively induced biomineralization to suppress osteosarcoma growth and metastases.

Biomineralization‐inducing polymers utilize the body's inherent Ca supply to form mineral layers, providing a prolonged therapeutic effect. The significant osteosarcoma suppression rates demonstrated the potential of these approaches to create effective barriers against tumor progression. However, the application of biomineralization in tumor treatment is not universally applicable to all tumor types and requires careful consideration of tumor biology. In certain tumor models, such as breast cancer, biomineralization may inadvertently create conditions within the TMEs that may facilitate metastases [32, 33]. Ca deposition, as a key aspect of biomineralization, influences the TMEs and tumor cell behaviors through various mechanisms. This process alters the chemical and physical properties of tumor tissue, promoting tumor cell proliferation and migration. Additionally, Ca deposition induces epithelial‐mesenchymal transition, enhancing the invasive potential of tumor cells. It may also facilitate the formation of new blood vessels within the tumor, further accelerating tumor growth. These factors highlight the important role of Ca deposition in driving metastases and tumor progression. To address these challenges, ongoing research is needed to understand the broader impact of biomineralization across different tumor types and develop strategies to mitigate its potential drawbacks.

## 3 Fibrogenesis

Fibrogenesis, similar to biomineralization, is a biological process in which fibrous protein aggregation alters tissue architecture and function [34, 35, 36]. While biomineralization involves the deposition of minerals by cells, fibrogenesis generally induces the self‐assembly of specific peptides or proteins into fibrous structures that significantly impact tissue structure and function [37]. These fibrous assemblies vary widely in morphologies, ranging from fibrils to larger fibers, depending on the biological context. Key features, such as their lengths, diameters, and degrees of cross‐linking, are critical in determining their mechanical properties and biological roles. A notable example is the pathological aggregation of amyloid‐*β* (A*β*) into fibrous plaques, a hallmark of Alzheimer's disease and other neurodegenerative diseases [38, 39]. Understanding these fibrous structures offers essential insights into assembly mechanisms that can be harnessed for developing therapeutic strategies, including constructing artificial tumor ECMs.

Building on this understanding, researchers have explored in situ self‐assembly strategies activated by pathological microenvironments [40, 41, 42, 43, 44]. Wang et al. devised a peptide that mimics laminin (LN), BP‐KLVFFK‐GGDGR‐YIGSR, which incorporates a fluorescent moiety (BP), a self‐assembling sequence (KLVFF), and a targeting sequence (RGD‐YIGSR) derived from LN [45]. Transmission electron microscopy (TEM) and scanning electron microscopy (SEM) images revealed structural changes in the peptide, which transformed from a spherical nanoparticle into a fiber‐like structure upon interaction with the tumor cell surface. When administered intravenously, this compound localized to the tumor site selectively, where it transformed into nanofibers, thus forming artificial tumor ECMs. This strategy resulted in significant reductions in lung metastases in models of both breast cancer and melanoma, achieving suppression rates of 82.3% and 50.0%, respectively. The ability of in situ self‐assembly of peptides to generate fibrous structures at a tumor site, thereby establishing an artificial ECM that acts as a physical barrier to tumor metastases, was demonstrated.

**FIGURE 2:**
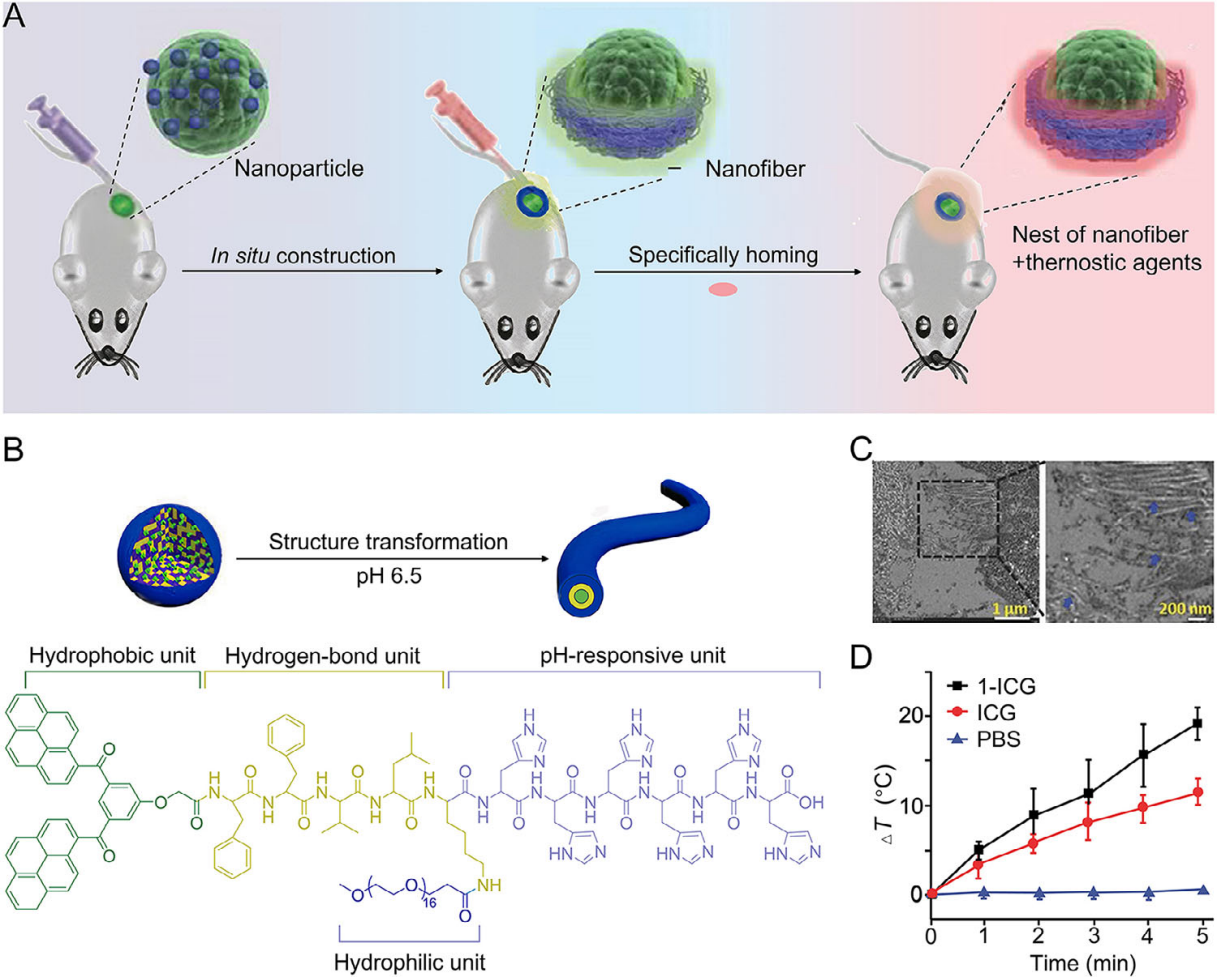
Constructing artificial tumor ECMs using a fibrogenesis strategy. (A) Pre‐settled nest‐like hosts in tumor for homing theranostics. (B) Morphology transformation from nanoparticle to nanofiber under acidic conditions. The molecular structure was designed with building blocks BP, KLVFF, His6, and PEG1000 motifs. (C) TEM images of tumor tissue slices of MCF‐7 xenografted mice *i.v*. injected with glycine labeled with iodine. (D) Temperature variations of tumor upon laser of MCF‐7‐bearing BALB/c nude mice. The mice were divided into three groups: (i) preconstructed NFs‐based nest‐like hosts, followed by ICG, (ii) only ICG, and (iii) only phosphate‐buffered saline (PBS) via *i.v*. injection (200.0 µL). Reproduced with permission [46]. Copyright 2017, John Wiley & Sons.

In addition to serving as physical barriers, these self‐assembled structures act as scaffolds that enhance the accumulation of therapeutic and diagnostic agents at the tumor site. In a subsequent study by Wang et al., fibrous assemblies were constructed at the tumor site, and fluorescent molecules or drugs were injected into mice. This approach enabled the targeted accumulation of therapeutic agents at the tumor site by leveraging hydrophilic, hydrophobic, and π–π interactions between small molecules and fibrous structures (Figure 2A−C) [46]. As a result, following injection of the near‐infrared dye indocyanine green (ICG), the nanoparticle‐treated group (1‐ICG) exhibited a higher temperature (55.7°C) than that treated with free ICG (49.6°C), indicating an enrichment of ICG that enhanced the photothermal effect (Figure 2D). This approach not only improved drug retention at the tumor site but also enhanced the efficacy of therapeutic intervention, showing significant potential for advanced tumor treatment.

Fibrogenesis‐based strategies for developing artificial tumor ECMs enable self‐assembling peptides to form fibrous structures at the tumor site, creating physical barriers that inhibit metastases and improve the localization and retention of therapeutic agents. However, despite its significant therapeutic potential, this approach encounters challenges, such as the enzymatic degradation of peptide fibers, which may limit long‐term effectiveness. Additionally, improving the specificity of these fibrous structures to minimize off‐targeted effects on healthy tissues is critical. Future research should focus on enhancing the stability and tumor‐specific targeting of fibrous ECMs to overcome these limitations. Strategies, such as incorporating *D*‐amino acids, which resist enzymatic degradation due to stereochemistry, significantly improve the overall efficacy of fibrous structures. *D*‐amino acids exhibit significant resistance to enzymatic degradation, making them less recognizable by proteolytic enzymes that typically target *L*‐amino acids. Furthermore, integrating these systems with other therapeutic modalities offers a more effective and targeted tumor treatment approach.

## 4 Gelation

In contrast to the fibrogenesis strategy that forms fibrous ECMs at the tumor site, gelation strategies create gel‐like structures by cross‐linking polymer biomaterials. These three‐dimensional (3D) networks closely mimic the natural ECMs of tumors. The gelation process can be achieved through physical or chemical cross‐linking. Recent efforts include using specifically designed polymers or harnessing natural gelation processes in the body to facilitate ECM formation.

**FIGURE 3:**
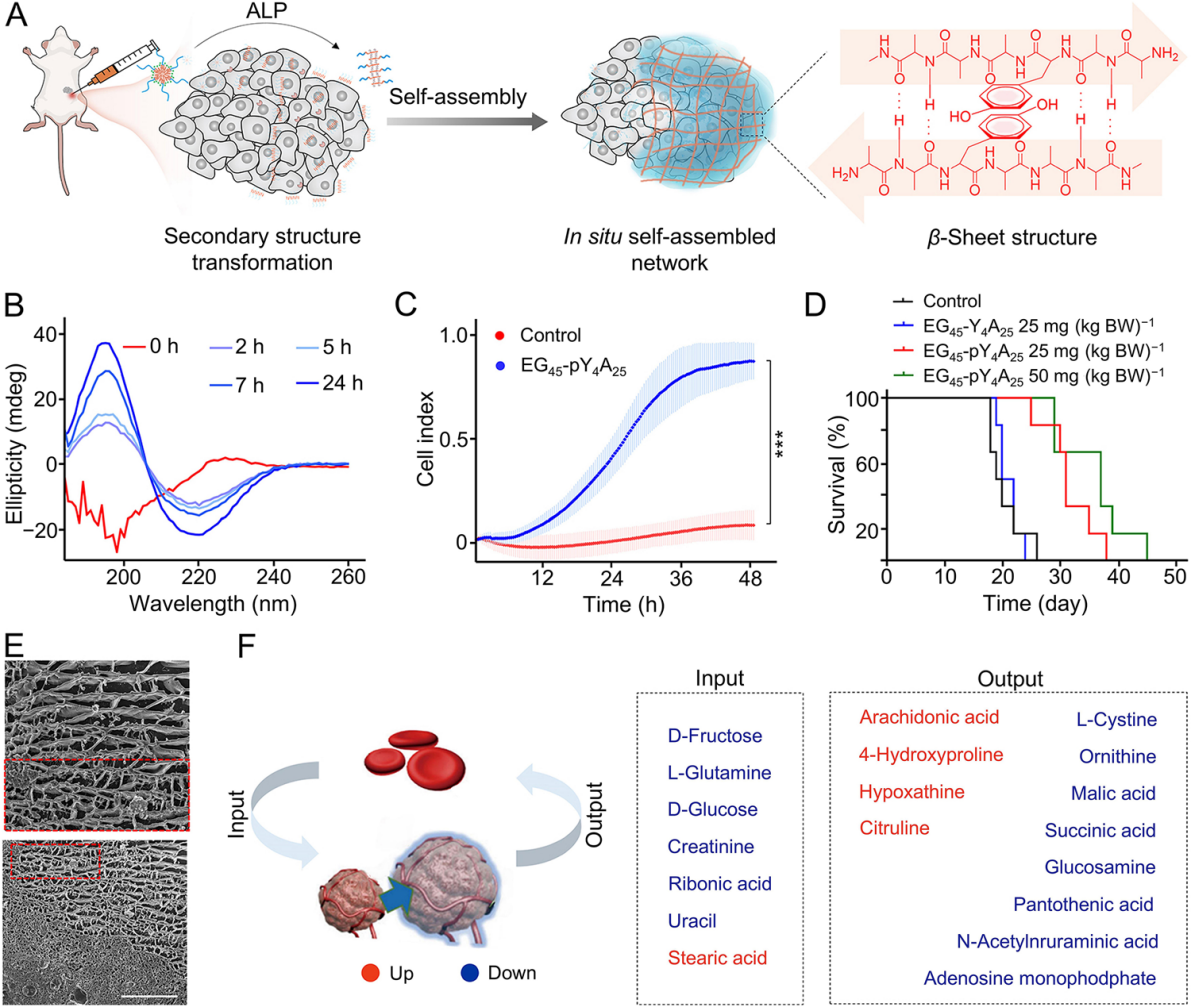
Constructing artificial tumor ECMs using a gelation strategy. (A) ALP‐inducing biomaterial underwent secondary structure transformation, further self‐assembled to form a network‐structured hydrogel that served as an artificial ECM. (B) Circular dichroism spectroscopy over time after adding ALP (0.5 IU mL^−1^) to 0.1 mg mL^−1^ of EG_45_‐pY_4_A_25_ solution at 37°C. (C) Analysis of 4T1 cell migration after treatment with PBS as a control and EG_45_‐pY_4_A_25_ at 0.01 mg mL^−1^ using a real‐time cell analysis (RTCA) system. (D) Survival rate of mice treated with saline as a control, EG_45_‐Y_4_A_25_ (25.0 mg (kg BW)^−1^), EG_45_‐pY_4_A_25_ (25.0 mg (kg BW)^−1^), and EG_45_‐pY_4_A_25_ (50.0 mg (kg BW)^−1^). (E) Cryo‐SEM images of tumor sections from mice treated with EG_45_‐pY_4_A_25_ at 50.0 mg (kg BW)^−1^. (F) List of input and output metabolites of tumors. All statistical data are represented as mean ± SD (*n* = 3; ***P < 0.001). Reproduced with permission [49]. Copyright 2024, Elsevier B.V. Reproduced with permission [52]. Copyright 2020, Springer Nature.

In gelation strategies, physical cross‐linking is often achieved by manipulating secondary structures. Poly(amino acid)s, such as poly(*L*‐alanine), which tend to form *β*‐sheet structures, often assemble into network structures [47, 48] and are commonly utilized as self‐assembly backbones. For example, Ding et al. designed and synthesized the poly(amino acid) methoxy poly(ethylene glycol)_45_‐*block*‐poly(*O*‐phospho‐*L*‐tyrosine_4_‐*co*‐*L*‐alanine_25_) (EG_45_‐pY_4_A_25_) (Figure 3A) [49]. Exploiting the high alkaline phosphatase (ALP) expression in the tumor site, the phosphoester bond was broken, changing the secondary structure from random coil to *β*‐sheet (Figure 3B). This transformation shifted EG_45_‐pY_4_A_25_ from nanoparticle to micron‐scale network structure, which induced apoptosis and inhibited tumor cell migration (Figure 3C). The artificial tumor ECMs mediated by EG_45_‐pY_4_A_25_ also effectively inhibited tumor progression, reduced metastases, and prolonged mouse survival (Figure 3D). As shown in Figure 3E, the biomaterial forms a 3D network structure with a layered arrangement at the tumor site, distinct from the dense structure of tumor tissue. Unlike conventional peptide self‐assembly that forms fiber, this study employs poly(amino acid) to induce self‐assembly of a network structure in situ. Large‐scale assemblies as artificial ECMs yield improved antitumor treatment efficacy. However, this network structure also faces challenges associated with enzymatic degradation, potentially limiting its long‐term effectiveness. Constructing *D*‐type network structures, which are more resistant to enzymatic breakdown, will solve this issue.

Another approach inspired by biological coagulation systems involves designing in situ forming hydrogels for artificial tumor ECM applications. In this process, fibrinogen in plasma is converted to fibrin by thrombin, creating a gel‐like network structure [50, 51]. Zhang et al. developed a method targeting fibrinogen modified with azide (Fb‐N_3_) to the tumor site with micro‐wounds [52]. Prothrombin grafted with dibenzocyclooctyne (Ptb‐DBCO) was subsequently used via bioorthogonal reactions to form fibrin hydrogels, acting as artificial ECMs. The cell behavior and metabolomics studies revealed that an artificial ECM impeded mass transport and induced tumor‐specific starvation (Figure 3F), inhibiting tumor growth by 77%. This physical barrier also prevented tumor cell metastases. However, micro‐wounds created by surgery, radiation therapy, or ultrasound treatments enhance gelation in normal tissues, necessitating thorough risk assessment.

The development of artificial tumor ECMs through gelation represents a significant advance in tumor therapy. Using physically cross‐linked poly(amino acid)s induced by *β*‐sheet formation, as well as the fibrin hydrogels, highlights the potential to improve tumor therapy by inhibiting tumor growth and metastases through physical barriers and tumor‐specific starvation. Looking ahead, several advancements are expected in artificial ECMs utilizing gelation strategies. First, enhancing biomaterial stability is crucial for ensuring long‐term effectiveness in the dynamic TMEs. Future research may focus on developing resilient cross‐linking methods or responsive biomaterials that adapt to biochemical changes. Second, increasing specificity to target only tumor tissues while sparing healthy cells involves tumor‐specific triggers, minimizing off‐target effects and enhancing precision. Finally, improving biocompatibility is essential to minimize immune responses and enhance the integration of these materials with host tissues.

## 5 Conclusion and Further Outlook

The ECMs play a crucial role in the formation and progression of tumors, serving as both a structural scaffold for tumor cells and a regulator of the TMEs through various biochemical signals, thereby accelerating the spread and invasion of tumor cells. Changes in the ECMs, such as alterations in the content and activity of structural and adhesion proteins, lead to biochemical and biomechanical modifications within the TMEs, thus affecting tumor malignancy. Current therapeutic research targeting tumor ECMs focuses on inhibiting matrix degradation to block metastases or promoting degradation to enhance the penetration of drugs or immune cells. However, these strategies may inadvertently release growth factors, triggering inflammation and tumor progression and possibly enhancing tumor migration and invasion.

Significant progress has been made in using biomaterials to construct artificial ECMs for tumor treatment. The biomineralization strategy leverages the body's inherent supply of ions to create lasting mineral layers, providing long‐term effects. The fibrogenesis strategy employs peptide self‐assembly to form fibrous structures that effectively concentrate drugs at the tumor site. Meanwhile, the gelation strategy closely mimics the complex 3D network of natural ECMs, enhancing the fit and function of artificial ECMs within the tumor. Together, these methods enhance tumor treatment effectiveness and offer tailored options based on specific tumor characteristics and therapeutic needs. While each strategy has its strengths, its differences lie in its mechanisms and applications (Table 1) [30, 31, 45, 46, 49, 52]. Biomineralization provides durability and stability but may apply to a narrower range of tumor types. Fibrogenesis excels in inducing assembly‐driven retention effects at the tumor site but may lack the structural complexity of gelation. Gelation, with its close imitation of natural ECM networks, provides superior adaptability and biocompatibility, though it may not achieve the long‐term stability provided by biomineralization. Understanding these similarities and differences allows researchers to tailor these strategies to specific tumor characteristics, maximizing the effectiveness of tumor blockade therapy.

**TABLE 1:** Biomaterials‐involved construction of extracellular matrices for tumor blockade therapy.

Extracellular matrix	Biomaterial	Mechanism	Tumor type	Therapeutic effect	Ref.
Biomineralization	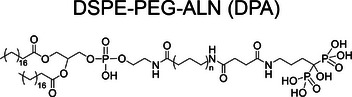	DSPE integrated into the membrane. The bisphosphonate group in ALN attracted Ca^2+^. Constructions of a calcified layer on tumor cell membrane.	Osteosarcoma	Biomineralized layer reduced substance exchange, acting as a barrier against metastases. ALN decreased osteoclast activity, inhibiting bone resorption and further preventing lung metastases.	[30]
	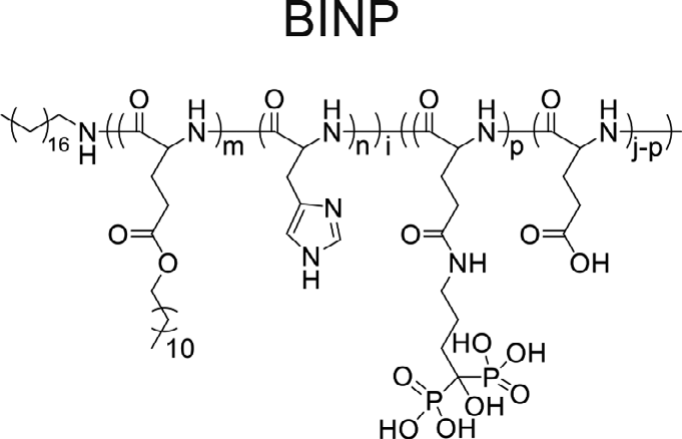	In acidic TMEs, the imidazole ring was protonized and transferred from hydrophobic to hydrophilic. BINP exposed dodecyl group for cytomembrane insertion. The bisphosphonate group attracted Ca^2+^, enabling biomineralization around the tumor tissue.	Osteosarcoma		[31]
Fibrogenesis	BP‐KLVFFK‐GGDGR‐YIGSR	The interaction between RGD and integrin facilitated the ligand‐receptor binding. A transition in secondary structure occurred, forming a β‐sheet structure. Nanoparticles are transformed into nanofibers through the establishment of ordered hydrogen bonds.	Breast tumor and melanoma tumor	Artificial ECMs served as a physical barrier against tumor metastases.	[45]
	BP‐KLVFFK‐PEG‐HHHHHH	Acidic microenvironments disrupted hydrophobic−hydrophilic balance. Increased proportion of *β*‐sheet structure. Transformation of nanoparticle into nanofiber.	Breast tumor	Nanofiber at the tumor site enhanced the accumulation of subsequently injected small molecules, such as drugs.	[46]
Gelation	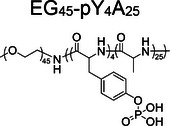	ALP catalyzed the cleavage of phosphate ester bonds, altering the biomaterial's hydrophobic–hydrophilic properties. Increase in β‐sheet structure proportion. Morphological transformation from nanoparticle to network‐structured hydrogel.	Breast tumor	The network structure functioned as a physical barrier to inhibit tumor metastases. The network structure contributed to the promotion of cell apoptosis.	[49]
	Azido‐modified fibrinogen (Fb‐N_3_) and azodibenzocyclooctyne‐grafted prothrombin (Ptb‐DBCO)	Micro‐wound from surgery prompted tumor‐specific accumulation. Ptb‐DBCO accumulated via bio‐orthogonal reaction. Altered vascular structures facilitated Ptb conversion to thrombin. Induced coagulation creates a reticular clot. Clot formed artificial ECM fibrin gel, entrapping the tumor.	Breast tumor and colon tumor	Artificial ECMs impeded mass transport, causing tumor‐specific starvation and growth inhibition. Artificial ECMs acted as a physical barrier against distant metastases in murine tumor model.	[52]

Although no clinical trials on artificial ECMs in tumor therapy are currently underway, their potential for clinical application is promising. However, several challenges must be addressed before clinical translation. First, biocompatibility and immune response are critical concerns for using artificial ECMs safely. To ensure long‐term safety, artificial ECMs must not trigger adverse immune reactions in vivo and must maintain compatibility with host tissues. Second, the controllable degradation of artificial ECMs is crucial for maintaining their therapeutic efficacy. In tumor therapy, the degradation times of biomaterials must be precisely regulated to ensure stable functionality during treatment, followed by safe elimination after therapy completion.

Additionally, the porosity of artificial ECMs must be optimized to prevent tumor cell migration while allowing efficient transport of therapeutic agents and supporting vascularization, thereby improving treatment outcomes. Further advancements include incorporating bioactive molecules, such as angiogenesis inhibitors, into artificial ECMs to deprive tumors of the nutrients and oxygen needed for tumor growth and metastases. Regarding biomaterial candidates, peptides and poly(amino acid)s have shown significant potential for use in artificial ECMs. These biomaterials exhibit excellent biocompatibility and offer controllable degradation properties, ensuring prolonged therapeutic effects while minimizing side effects. Peptides and poly(amino acids) have shown significant potential as biomaterials for artificial ECMs, exhibiting excellent biocompatibility and controllable degradation properties, which ensure prolonged therapeutic effects while minimizing side effects. Future research should focus on refining these biomaterials to optimize their performance and facilitate their entry into clinical trials. In summary, while challenges remain in bringing artificial ECMs into clinical use, continuous improvements in biomaterial performance, safety, and functionality are promising for advancing artificial ECMs into clinical use. These advances offer new therapeutic hope for cancer patients, potentially revolutionizing the treatment landscape.

## Author Contributions

**Jinfeng Sun**: conceptualization, investigation, resources, writing – original draft, writing – review and editing. **Yang Liu**: writing – review and editing. **Jingshan Sun**: writing – review and editing. **Jianxun Ding**: conceptualization, writing – review and editing, supervision, project administration, funding acquisition. **Xuesi Chen**: conceptualization, writing – review and editing, supervision, project administration, funding acquisition.

## Conflicts of Interest

The authors declare no conflicts of interest. Xuesi Chen and Jianxun Ding are members of the *Exploration* editorial board, and they were not involved in the handling or peer review process of this manuscript.
